# Differences in the Composition of Gut Microbiota between Patients with Parkinson’s Disease and Healthy Controls: A Cohort Study

**DOI:** 10.3390/jcm10235698

**Published:** 2021-12-03

**Authors:** Barbara Zapała, Tomasz Stefura, Magdalena Wójcik-Pędziwiatr, Radosław Kabut, Marta Bałajewicz-Nowak, Tomasz Milewicz, Alicja Dudek, Anastazja Stój, Monika Rudzińska-Bar

**Affiliations:** 1 Department of Clinical Biochemistry, Jagiellonian University Medical College, 31-066 Krakow, Poland; 2 2(nd) Department of General Surgery, Jagiellonian University Medical College, 31-008 Krakow, Poland; tomasz.stefura@gmail.com (T.S.); ala.ddudek@gmail.com (A.D.); 3 Department of Neurology, Andrzej Frycz Modrzewski Krakow University, 30-705 Krakow, Poland; m.pedziwiatr@szpitaljp2.krakow.pl (M.W.-P.); mrudzinska@afm.edu.pl (M.R.-B.); 4 Institute of Psychology, Jagiellonian University, 30-060 Krakow, Poland; radoslaw.kabut@gmail.com; 5 Department of Gynecology and Obstetrics, Jagiellonian University Medical College, 31-501 Krakow, Poland; marta.balajewicz@gmail.com; 6 Department of Gynaecological Endocrinology and Gynaecology, Jagiellonian University Medical College, 31-501 Krakow, Poland; tomasz.milewicz@uj.edu.pl; 7 Department of Hematology Diagnostics and Genetics, The University Hospital, 30-688 Krakow, Poland; astoj@sukrakow.pl

**Keywords:** Parkinson’s disease, microbiota, gut microbiome

## Abstract

Gut microbiome and colonic inflammation can be associated with the predisposition and progression of Parkinson’s disease (PD). The presented study aimed to compare gastrointestinal microbiota composition between patients diagnosed with PD and treated only with Levodopa to healthy controls. In this prospective study, patients were recruited in 1 academic hospital from July 2019 to July 2020. The detailed demographic data and medical history were collected using a set of questionnaires. Fecal samples were obtained from all participants. Next-Generation Sequencing was used to assess the microbiota composition. The endpoint was the difference in composition of the gut microbiota. In this study, we enrolled 27 hospitalized PD patients with well-controlled symptoms. The control group included 44 healthy subjects matched for age. Among PD patients, our results presented a higher abundance of *Bacteroides* phylum, class *Corynebacteria* among phylum *Actinobacteria*, class *Deltaproteobacteria* among phylum *Proteobacteria*, and genera such as *Butyricimonas, Robinsoniella*, and *Flavonifractor*. The species *Akkermansia muciniphila*, *Eubacterium biforme*, and *Parabacteroides merdae* were identified as more common in the gut microbiota of PD patients. In conclusion, the patients diagnosed with PD have significantly different gut microbiota profiles in comparison with healthy controls.

## 1. Introduction

Parkinson’s disease (PD) is the second most common neurodegenerative disease worldwide, with a prevalence of 1% in the population above 60 years [1]. This progressive disease is diagnosed based on clinical symptoms observations such as resting tremor, bradykinesia, rigidity, and postural instability [2]. PD is characterized as alpha synucleinopathy that propagates through the brain-gut axis from the autonomic, enteric nervous system to the brain. 

In 2004, Sudo et al. described a bidirectional communication system involving neural and humoral mechanisms [3]. In this experiment, the authors identified the impaired stress response in germ-free mice. Recent studies confirmed the meaning of the gut-brain axis, which consists of anatomical neural connections, humoral components provided by both the endocrine and immune systems, and the intestinal epithelium and the gut microbiome [4,5]. The results strongly suggested that this bidirectional connection between the central nervous system and gut is mainly regulated by gut microbiota. The gut microbiota influences the gut-brain axis primarily by immunological, neuroendocrinal, and direct neural mechanisms [6,7].

As mentioned, colonic inflammation was identified in over 80% of PD cases, and they precede the motor symptoms even by several years [8]. Therefore, there is much evidence emphasizing the role of the gut microbiome and colonic inflammation in the predisposition and progression of PD. At least ten studies have been performed in which the differences in gut microbiota composition in patients with PD were detected [9,10,11,12,13,14,15,16,17,18,19]. However, in these studies, there were differences in patient enrollment and methods of analysis. When looking at the results obtained by using 16S rRNA sequencing, the most abundant bacteria in patients with PD in comparison to healthy subjects were: *Firmicutes*, *Proteobacteria*, *Verrucomicrobia*, and *Actinobacteria*, whereas the less abundant were *Bacteroidetes* [9,10,12,13,15,16,17,18,19].

The presented study aimed to compare the composition of gastrointestinal microbiota composition between patients diagnosed with PD and treated only with Levodopa to healthy controls.

## 2. Materials and Methods

### 2.1. Study Design

According to motor symptoms assessment, patients were clinically classified using Hoehn & Yahr staging (H&Y) and Movement Disorders Society Unified Parkinson’s Disease Rating Scale part III (MDS-UPDRS Part III) score during ON time to estimate disease progression and motor impairment. Patients were recruited in one academic hospital from July 2019 to July 2020. Both the cognitive and functional status were scored using the Montreal Cognitive Assessment (MoCA). The detailed demographic data and medical history were collected using a set of questionnaires. The exclusion criteria included: other neurologic diagnoses, systemic or neurologic infections, inflammatory or autoimmune diseases, atypical parkinsonism syndrome and vascular parkinsonism, brain surgery, concomitant psychiatric diseases such as schizophrenia or bipolar disorder, preliminary baseline evaluations, confirmed or suspected gastrointestinal malignant tumor or other gastrointestinal diseases, the use of antibiotics or probiotics over the last three weeks or therapy based upon steroids, non-steroidal anti-inflammatory drugs, history of gastrointestinal surgery (e.g., gastro-resection or major intestinal surgery). We enrolled the control group in 44 healthy subjects matched for age and without any medical history for neurological, immunological, and gastrointestinal diseases. 

### 2.2. Biological Samples Collection

About 1–2 g of fresh fecal samples were collected in the sterile tubes and stored at −80 °C until the subsequent preparation. Bacterial genomic DNA was extracted from 250 mg of homogenized feces using QIAamp PowerFecal Pro DNA Kit (QIAGEN, Hilden, Germany). All of the steps during the extraction were performed according to the manufacturer’s instructions. The amount of extracted DNA was quantified and qualified using spectrophotometer NanoDrop ND-1000 (Thermo Electron Corporation, Waltham, MA, USA) and fluorometer Qubit 4 (Invitrogen, Waltham, MA, USA). Then, all DNA samples were stored at −20 °C until further analysis.

### 2.3. Genetic Library Construction

The libraries were constructed using gene-specific sequences targeting the V3 and V4 regions of the 16S rRNA gene. These primers were adapted from the Klindworth et al. publication [20]. Libraries were prepared in accordance with the protocol for Preparing 16S Ribosomal RNA Gene Amplicons for Illumina MiSeq System (Part#15044223Rev.B). According to the manufacturer’s recommendations, the PCR-based amplification was performed using KAPA HiFi HotStart ReadyMix (ROCHE, Basel, Switzerland). The thermocycling profile of the amplification was as follows: 95 °C for 1 min, 55 °C for 1 min, then 72 °C for 1 min for 30 cycles, followed by a final extension at 72 °C for 5 min. Amplicons were then indexed with specific sequencing adapters using the Nextera XT Index Kit v2 from Illumina. The program of indexing PCR on a thermal cycler was as follows: 95 °C for 3 min, eight cycles of 95 °C for 30 s, 55 °C for 30 s, 72 °C for 30 s, followed by a final extension at 72 °C for 5 min and hold at 4 °C. Before sequencing, the integrity and size (~630 bp) of amplicons were determined on Qubit 4.0 Fluorometer (Invitrogen) and Bioanalyzer (Agilent, Santa Clara, CA, USA) using Bioanalyzer DNA 1000 chips. Then amplicons were pooled in equimolar concentrations and sequenced on MiSeq instrument (Illumina, San Diego, CA, USA) using a 300 × 2 V3 Kit and PhiX Control V3 from Illumina.

### 2.4. Bioinformatic and Statistical Analysis

The raw data of 16S rRNA gene sequences generated as FASTQ files from the MiSeq run were first classified using the Illumina16S Metagenomics workflow. The classification was performed based on the algorithm with the high-performance implementation of the Ribosomal Database Project (RDP) classifier, described by Wang Q. et al. in 2007 [21]. Then, the open-reference operational taxonomic unit (OUT) tables were prepared, and the downstream analysis was performed. The taxonomic assignment of individual datasets was prepared using Greengenes database version 13.5 [22]. Alpha and beta diversity were calculated using QIIME 2.0 software with Python scripts [23]. Alpha diversity was calculated based on the sequence similarity at the level of 97%. The richness was calculated as the amount of unique OTUs found in each sample and presented as observed OTUs, and the count of unobserved species based on low-abundance OTUs was presented as ACE and Chao1 indices. Shannon, Simpson, and Fisher estimators were calculated to measure both the richness and evenness within individual samples and in the experimental groups of samples [24,25]. Beta diversity (the distance and dissimilarities in-between microbial communities) was determined based on Jaccard, Bray-Curtis, Jensen-Shannon Divergence indices calculated by QIIME. The distances were visualized by principal coordinate analysis (PCoA) [26]. 

The following clustering and further statistical analysis were performed with LEfSe [27] and MicrobiomeAnalyst platform [28]. The characteristic features of intestinal microbiota profiles were determined using the linear discriminant analysis (LDA) effect size with LEfSe. By using LEfSe, we described the biomarkers with the highest statistical and biological significance. The discovered microbial biomarkers with statistical significance and biological relevance were described based on the normalized relative abundance matrix, the Kruskal-Wallis rank-sum test, the significant alpha at 0.05, and the effect size threshold of 2. The hierarchical structure of taxonomic classifications was characterized using the median abundance and the non-parametric Wilcoxon Rank Sum test to show the taxonomic differences between the microbial communities and the abundance profiles of two experimental groups [29].

Statistical analysis was performed using the SPSS v19.0 (Chicago). Continuous variables were calculated using non-parametric, *t*-test, and Mann-Whitney *U*-test. Categorical data were calculated using Pearson chi-square or Fisher tests. Spearman’s rank correlation test was applied to perform correlation analyses. 

## 3. Results

### 3.1. Subject Characteristics

In this study, we enrolled 27 hospitalized PD patients with well-controlled symptoms. The baseline characteristics of PD patients and age-matched healthy controls (44 volunteers) are shown in Table 1. The mean age of PD patients was 67 years and of healthy controls was 64 years. There were no significant differences in sex, smoking, drinking, and ethnicity between the PD patients and healthy controls (*p* > 0.05). The MoCA, H&Y, and MMSE scores were low in PD patients (Table 1). 

Among PD patients, 12 (46.15%) were diagnosed with hypertension, 9 (34.62%) with coronary disease, and 6 (23.08%) with diabetes mellitus type 2. Blood parameters did not show any significant differences between the two groups. Among the lipid parameters such as triglyceride, T-cholesterol, high-density lipoprotein cholesterol, and low-density lipoprotein cholesterol, there were no significant differences between the two groups, and their levels were in the normal ranges (Table 2). Table 2 also shows the characteristics of urine samples in which there were no abnormalities observed, except urine color (changed in 3 patients with PD) clarity (changed in 5 PD patients) and bacteria present in 5 urine samples of patients with PD (Table 2). 

Based on CT/MRI in several patients, cerebrovascular disease is a subcortical white matter disease. Additionally, symmetric atrophy in the frontal and parietal lobes on the structural brain was observed using MRI. 

### 3.2. The Overall Characterization of the Run Parameters and Structure of Fecal Microbiota

A total of 5,949,989 high-quality reads, with an average of 94,444 reads per sample, were obtained to analyze the microbiota. The coverage was 99.9%, which indicated that most bacterial phylotypes (3466 OTUs) in the fecal microbiota had been identified. 

The profiles of PD patients’ and controls’ fecal microbiota differed significantly, and the alpha and beta diversity differed in the two studied groups. 

**Figure 1:**
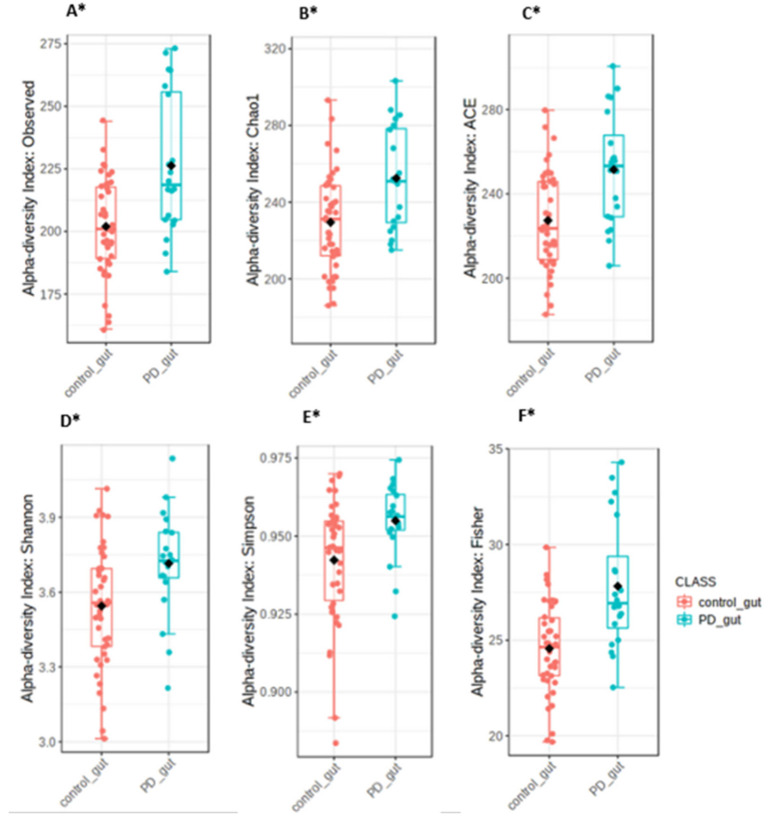
The richness indices of the observed OTUs (**A**), Chao1 (**B**), and ACE (**C**), and the diversity indices of Shannon (**D**), Simpson (**E**), and Fisher (**F**), showing the overall structure of the fecal microbiota in PD patients and healthy controls. The symbol * represents the statistical significance * *p* < 0.05.

The alpha-diversity indices, including Shannon’s (*p*-value < 0.007), Simpson’s (*p*-value: 0.003), and Fisher’s (*p*-value < 0.001), were significantly different between PD patients and controls. In PD patients, significantly higher bacterial diversity was observed than in the controls. The richness based on observed OTUs (*p*-value < 0.002), ACE (*p*-value < 0.001), and Chao1 (*p*-value < 0.003) indices were significantly higher in PD patients than in controls, as shown in Figure 1.

**Figure 2:**
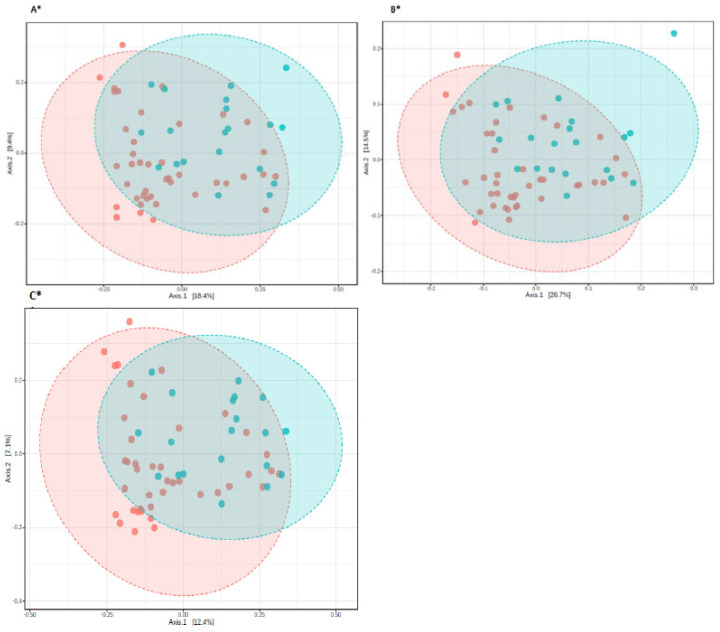
Principal coordinate analysis (PCoA) plots of individual fecal microbiota based on Bray-Curtis (**A**), Jensen-Shannon Divergence (**B**), Jaccard Index (**C**). Red dots represent controls, while blue ones represent PD patients. The symbol * represents the statistical significance * *p* < 0.01.

The beta diversity demonstrated as PCoA based on the Bray–Curtis, Jensen-Shannon, and Jaccard, an algorithm indicating the statistical difference between PD patients and controls (*p*-value < 0.01). Figure 2 presents the beta diversity in study groups.

### 3.3. The Microbiota Characteristics

**Figure 3:**
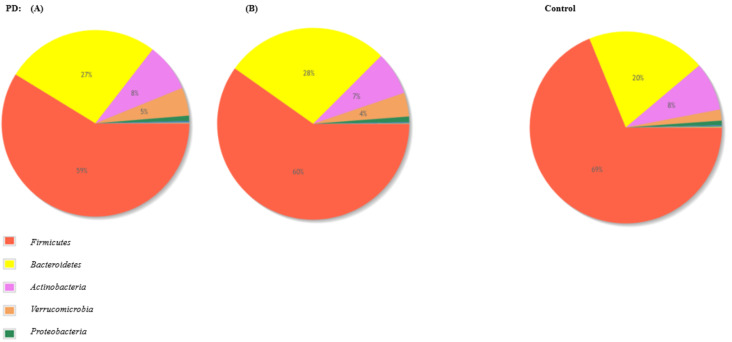
Pie charts presenting the comparative abundance profiles across two groups PD and control at the phylum level. On the left, pie chart (**A**) represents the study group diagnosed with PD and other diseases (such as hypertension and diabetes), the pie chart (**B**) represents the study group diagnosed with PD and without other diseases.

The gut microbiota among PD patients was mainly composed of bacteria from phyla *Firmicutes* (59%), *Bacteroidetes* (27%), and *Actinobacteria* (8%). Moreover, the participants of the control group presented gut microbiota composed of phyla *Firmicutes* (69%), *Bacteroidetes* (20%), and *Actinobacteria* (8%). Charts presenting pooled microbiota composition in terms of phyla in both groups are presented in Figure 3.

**Figure 4:**
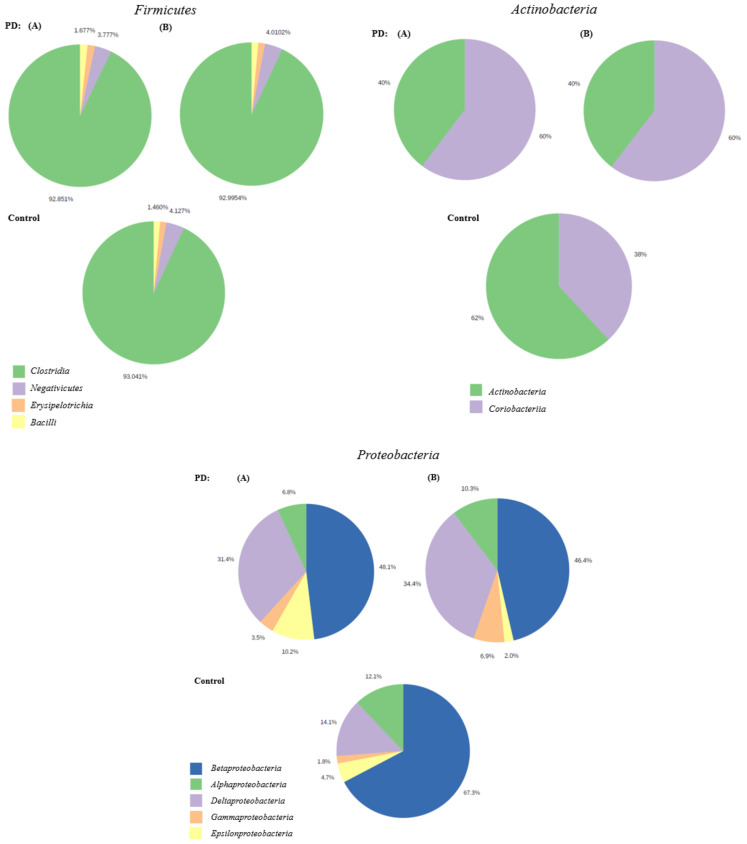
Pie charts presenting the comparative abundance profiles across two groups PD and control at class level in the phyla *Firmicutes*, *Actinobacteria*, and *Proteobacteria*. Pie charts with (A) represent the study group diagnosed with PD and other diseases (such as hypertension and diabetes), the pie charts with (B) represent the study group diagnosed with PD and without other diseases.

Among the patients diagnosed with PD, class *Clostridia* was the most common in phylum *Firmicutes* (92.39%), class *Coriobacteriia* was the most abundant in phylum *Actinobacteria* (60%), and class *Betaproteobacteria* was most common in phylum *Proteobacteria* (48.1%). Class *Clostridia* was the most common among phylum *Firmicutes* in the control group, class Actinobacteria (60%) was the most abundant in phylum *Actinobacteria*, and class *Betaproteobacteria* was the most abundant the most common in phylum *Proteobacteria*. Charts presenting pooled microbiota composition in terms of class in phyla *Firmicutes*, *Actinobacteria*, and *Proteobacteria* in both groups are presented in Figure 4.

**Figure 5:**
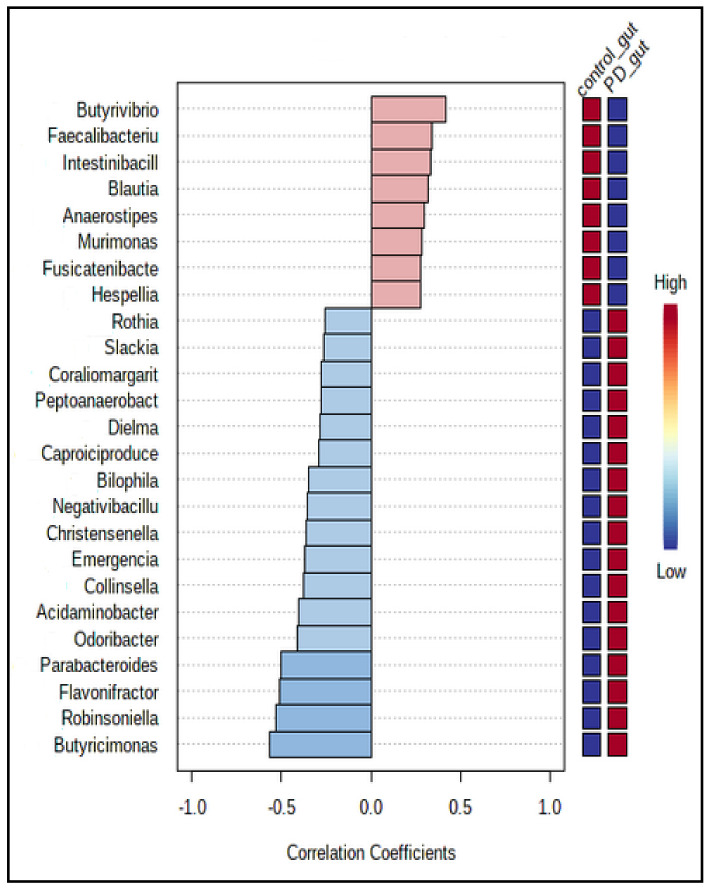
The Pearson *r* correlation, showing the top 25 features correlated with the taxa of interest. The 25 genera were ranked by their correlation. The blue color represents negative correlations, whereas the red color represents positive correlations. The deeper color (blue or red) means, the stronger correlation. The mini heatmap on the right side of the plot shows the high or low abundance in two groups.

Figure 5 presents 25 genera of bacteria most strongly correlated with the patients’ PD/control group allocation. For instance, *Butyricimonas*, *Robinsoniella*, and *Flavonifractor* correlated positively with PD allocation, whereas *Butyrivibrio*, *Faecalibaculum*, and *Intestinibacillus* correlated most negatively with this group.

**Figure 6:**
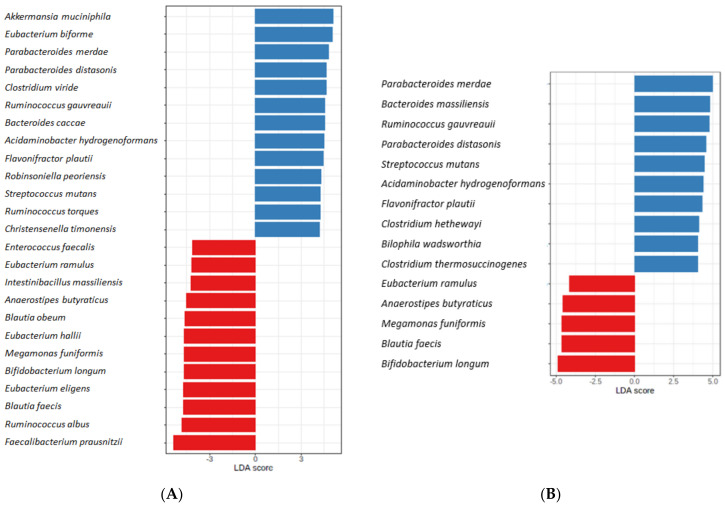
Bacterial species are more abundant in the PD than in the control group, according to the LefSE analysis. Features, as species, are described on the left side of the graphical output presented as bar plots. Red bar plots represent healthy controls and blue—PD patients. The presented features were selected based on the *p*-values < 0.05. (**A**) with bar plot represents the results of the study group with PD and other diseases (such as hypertension and diabetes), and (**B**) represents the results of the study group diagnosed only with PD, without other diseases.

The analysis identified multiple species of bacteria, which were more abundant in each study group, as presented in Figure 6. For instance, *Akkermansia muciniphila*, *Eubacterium biforme*, and *Parabacteroides merdae* were significantly more common among PD patients. Whereas *Faecalibacterium prausnitzii*, *Ruminococcus albus*, and *Blautia faecis* were, among other things, more abundant among participants of the control group.

**Figure 7:**
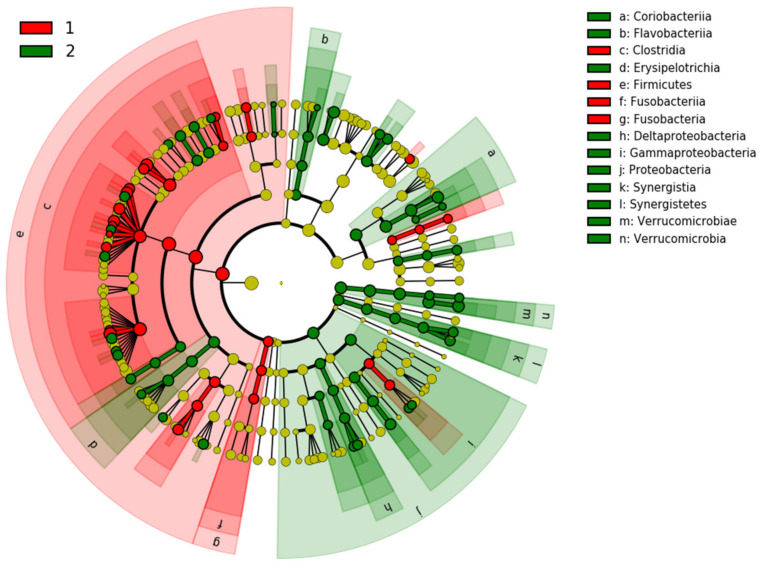
Cladogram generated by LEfSe shows the taxa differences between PD patients (2) and healthy controls (1). The central yellow dot in each cladogram represents kingdom; each successive circle is one step lower phylogenetically (phylum, class, order, family, and OTU). Regions colored in red indicate taxa enriched in controls compared to those enriched in PD patients marked with green regions.

Cladogram generated by LEfSe and shown in Figure 7 demonstrates that samples of PD were enriched for *Coriobacteriia*, *Flavobacteriia*, *Erysipelotrichia*, *Deltaproteobacteria*, *Gammaproteobacteria*, and *Verrucomicrobia*, whereas samples from healthy controls were primarily enriched in *Clostridia*, *Firmicutes*, and *Fusobacteria*.

**Figure 8:**
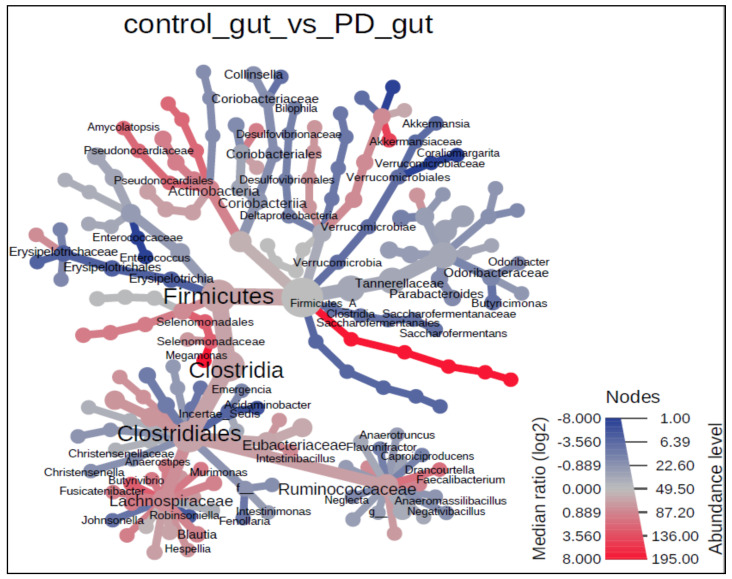
The heat tree analysis, showing the taxonomic differences of fecal microbiota between PD and healthy controls. Only significant taxon names were labeled on the heat tree. Statistical significance was calculated using the non-parametric Wilcoxon Rank Sum test with 0.05 Wilcoxon *p*-value cut-off.

Bacterial taxa, whose abundance was significantly different between PD patients and the control group, is displayed as a heated tree included in Figure 8. This figure represents observed significant differences on various taxonomic levels.

## 4. Discussion

Our study attempted to reveal the potential relationship between gastrointestinal microbiota and PD. We decided to use a cutting-edge method for microbiota composition analysis—Next Generation Sequencing, which allows us to assess all bacterial genetic material present in each sample and identify present bacterial species. Therefore, we believe that this study provides new insight into the research concerning the pathogenesis of PD and certainly is another step towards understanding gut microbiota’s role in developing and managing PD symptoms.

Pathological, PD is characterized by the formation of insoluble alpha-synuclein (αSyn) aggregates within neurons, which contributes to the loss of dopaminergic neurons in the basal ganglia [30,31]. This pathology also occurs more widely throughout the central and peripheral nervous systems, including the enteric nervous system and the gastrointestinal tract [32]. 

Although PD is primarily recognized as a movement disorder, there is a range of associated nonmotor symptoms, including cognitive impairment, depression, and sleep disturbances [33]. These can occur throughout the disease course, even predating the motor syndrome [33]. The non-motor symptoms are thought to be associated with dysfunction of the gastrointestinal tract, and the disease is recognized as not only the disease of the brain [33,34,35]. The studies performed in the past years strongly suggest the pivotal role of inflammation and alterations in the gut microbial communities in patients with PD [33,34,35]. These results indicated that non-motor symptoms in patients with PD are strongly associated with dysfunction of the microbiota-gut-brain axis [33].

The human gut microbiota consists of 1014 resident microorganisms, including bacteria, archaea, viruses, and fungi [34]. It shows a high degree of variability among individuals, reflecting differing exposures to environmental factors and the influence of host phenotype such as age and ethnicity [35]. It has been shown the four phyla thatprimarily dominate the gut bacteria in healthy individuals are *Actinobacteria*, *Firmicutes*, *Proteobacteria*, and *Bacteroidetes* [35]. The large intestine is the site of the most significant number and diversity of bacterial species, with recent estimates of 1011 bacteria per gram of colon contents [36,37]. The gut microbiota is strongly influenced by habitual diet [38]. Both aging and the presence or absence of disease conditions influence the microbiota composition [39]. Multiple reports show the involvement of abnormal gut microbiota, termed dysbiosis, which has been seen in many pathological conditions such as obesity and individuals with chronic age-related conditions [39,40,41,42,43].

Previous studies concerning the role of gut microbiota in PD revealed multiple potential correlations. According to Keshavarzian et al., gut-dysbiosis can induce a pro-inflammatory reaction, resulting in misfolding of α-Syn and inducing the development of PD [44]. A study by Scheperjans et al. has revealed a reduced number of *Prevotellaceae* families in the feces of PD patients [9]. At the same time, the *Enterobacteriaceae* family was positively associated with the severity of postural instability and gait difficulty [9]. Increased intestinal permeability seems to be strongly correlated with increased exposure to endotoxin and oxidative stress burden in the intestine, which is associated with abnormal accumulation of α-Syn and development of PD [9]. Previous studies concerning the role of gut microbiota in PD revealed multiple potential correlations. According to Keshavarzian et al., gut-dysbiosis can induce a pro-inflammatory reaction, resulting in misfolding of α-Syn and inducing the development of PD [44]. A study by Scheperjans et al. has revealed a reduced number of *Prevotellaceae* families in the feces of PD patients [9].

In comparison, the *Enterobacteriaceae* family was positively associated with the severity of postural instability and gait difficulty [9]. Increased intestinal permeability seems to be strongly correlated with increased exposure to endotoxin and oxidative stress burden in the intestine, which is associated with abnormal accumulation of α-Syn and development of PD [9]. In this study, we showed significant alterations in the gut microbiota composition in PD patients either diagnosed with associated comorbidities or not. PD patients with or without associated comorbidities have a more significant abundance of the *Bacteroides* phylum. Additionally, *Coriobacteriia*, *Deltaproteobacteria*, and genera such as *Butyricimonas*, *Robinsoniella*, and *Flavonifractor* were more common in the PD group. According to Figure 3, the gut microbiota of PD patients was more abundant, especially in *Bacteroidetes* and *Verrucomicrobia*. Those results suggest a significant decrease in the abundance of bacteria with pro-inflammatory species and a significant decrease in the abundance of the bacteria with anti-inflammatory properties. Understanding the role of gut microbiota in their pathology may utilize this knowledge in the diagnosis, treatment, and prevention of these diseases. In 2021, the meta-analysis article was published in which the authors applied a standardized workflow to analyze each study concerning gut microbiome, individually and they described the significant changes affecting the gut microbiome of PD patients across sampling cohorts [45]. According to this report, in PD patients a lower abundance of genera such *Roseburia*, *Fusicatenibacter*, *Blautia*, *Anaerostipes* (*Lachnospiraceae* family), and *Faecalibacterium* (*Ruminococcaceae* family) should be observed [45]. These taxa are abundant and widespread bacteria in the gut microbiota of healthy individuals, they are major butyrate producers, and it has been shown that a lower level of these taxa can influence an intestinal epithelial barrier permeability and initiate an inflammation which is linked with PD and observed gastrointestinal symptoms related to the disease [9,46]. Comparable to the other authors, in this study, we also demonstrated the decrease in *Faecalibacterium*, *Fusicatenibacter*, and *Blautia* in PD patients. As reported by others, these taxa are correlated with gut inflammation [47]. 

In this study, in patients with PD and other comorbidities, the most abundant species was *Akkermansia muciniphila*, and in patients diagnosed only with PD, it was *Parabacteroides merdae* which accords with the data from the literature. *Akkermansia muciniphila* is mucin-degrading bacteria enriched in neurodegenerative diseases (such as Alzheimer’s disease or multiple sclerosis) and contributes to the progression of neural pathologies by degrading mucin increasing gut inflammation and permeability, and finally leading to higher endotoxemia and systemic inflammation [46,47]. Even in animal studies, it has been demonstrated that *Akkermansia* is responsible for the depletion of the intestinal mucus layer, dried stools, decreased goblet cells, and impaired intestinal barrier function [48,49,50,51,52]. In this study, we observed the higher abundance of *A. muciniphila*, *Eubacterium* sp., and the *Clostridiales* family in patients with PD. This aligns with the literature which 388 shows bacteria is the primary producer of homocysteine [49,50]. The decrease in *Prevotellaceae* was observed in PD patients, which has also been previously described as contributing to the alterations in the enteric nervous system in PD patients [53]. The microbial profile discovered in this study may be a consequence of PD progression and disease treatment. However, it may be strongly related to high mucus degradation resulting in gut permeability, which is linked to the non-motor symptoms of the disease observed, especially at the onset of the disease. To date, a limited number of bacterial species from the *Bacteroidetes*, *Firmicutes*, *Actinobacteria*, and *Verrucomicrobia* phyla have been studied for their ability to consume mucins [54]. The most studied were Bacteroidetes which showed the highest ability to consume mucins. In the phylum of Firmicutes, some species such as *Ruminococcus torques* and *Ruminococcus gnavus* have also been shown to degrade mucins and use them as sole carbon sources [53]. The *Verrucomicrobia* phylum, *Akkermansia muciniphila*, was reported as a strictly anaerobic Gram-negative bacterial species and a key mucin degrader [54,55]. Our results suggest that gastrointestinal, non-motor symptoms maybe not be secondary to PD progression but rather one of the mechanisms involved in the etiology of the disease.

This study is associated with several limitations. Firstly, the study group was relatively small and recruited only in one center. Unfortunately, due to limited funding findings and the high cost of conducting NGS analyses, we limited the sample size. The study group included both patients diagnosed with PD and associated comorbidities which limited the final results. Comorbidities associated with PD (such as diabetes, anemia, hypertension, and others) have important implications in the health outcomes and clinical management of PD patients [56].

Moreover, as reported, these comorbidities might appear even before the onset of PD and thus play a pivotal role as risk factors for PD [57]. Nevertheless, we have obtained statistically significant results allowing us to conclude. The generalizability of these results is limited and should be conducted with caution. Secondly, the nature of the study was comparative, and it is difficult to identify cause and effect. Moreover, the precision of the results in this type of study is limited by the sequencing efficiency. We did, however, include a large number of sequences in presented microbiota analyses.

Since 2014, several large-scale case-control studies have shown significant changes in the gut microbiota composition [58]. In all of these studies, it was very problematic to establish the composition changes at the phylum level due to the high variability in the relative abundances of bacteria phyla in patients with PD. It remains unclear how the gut microbiome changes during PD and whether different microbes are related to different phenotypes of PD.

The results of this report aligned with the literature and even shed more light on gut microbial dysbiosis in patients with PD. We characterized the gut microbiome at the species levels in PD patients with idiopathic disease. Moreover, the study group had a similar clinical phenotype, without stupor. MMSE scores were about 27 and Hoehn & Yahr about 2. All PD patients were undiagnosed with autonomic symptoms and were of the same ethnicity.

The results presented in this report have a strong meaning in managing and especially in the treatment of patients with PD. Discovering the bacteria involved in dysbiosis between enteric pathogens and commensal mucin-degrading, butyrogenic bacteria or bacteria with proved enzymatic activity, we show the significance of enteric infection and gut dysbiosis on the pathogenesis of PD and demonstrate the potential of using these bacteria as a preventative and treatment strategy for the disease.

Further research should be conducted on a more extensive study group recruited in multiple centers. It would also be beneficial to correlate specific symptoms or subtypes of PD with specific microbiota profiles. In the long term, we are hoping to develop an intervention aimed at modifying microbiota to achieve optimal management of PD symptoms or prevent the development of PD in the first place. 

## 5. Conclusions

This study is the first to characterize the gut microbiota profiles in Polish patients with PD and associated comorbidities and treated with Levodopa. Patients diagnosed with PD have significantly different gut microbiota profiles in comparison with healthy controls. We observed a more significant abundance of *Bacteroides* phylum among PD patients, and class *Corynebacteria* among phylum *Actinobacteria* and class *Deltaproteobacteria* among phylum *Proteobacteria* were more abundant in this group than the controls. Genera such as *Butyricimonas*, *Robinsoniella*, and *Flavonifractor* positively correlated with allocation to the PD group. Additionally, species such as *Akkermansia muciniphila*, *Eubacterium biforme*, and *Parabacteroides merdae* were identified as more common in the gut microbiota of PD patients.

## Figures and Tables

**Table 1:** Baseline characteristics of the study groups, PD and healthy controls. Mean ± SD or *n* (%).

Parameter	PD, *n* = 27	Healthy Controls, *n* = 44
Age	67.63 ± 8.15	64.36 ± 7.12
Hoehn & Yahr score	2.04 ± 0.71	-
Montreal Cognitive Assessment scale	21.8 ± 5.15	-
Mini-Mental State Examination	26.33 ± 1.52	-
Duration of Parkinson’s disease	7.32 ± 6.67	-
Levodopa dosage	554.35 ± 277.13	-
Family history of Parkinson	2 (7.69)	negative
Hypertension	12 (46.15)	not diagnosed
Heart disease	9 (34.62)	not diagnosed
Diabetes mellitus	6 (23.08)	not diagnosed
Degeneration of the spine	5 (19.23)	not diagnosed

**Table 2:** Serum biochemical parameters and some other characteristics of urine samples of a patient with PD and healthy controls.

Parameter	PD Patients (*n* = 27) Mean ± SD	Healthy Controls (*n* = 44) Mean ± SD
**Blood**		
White blood cells [Thous/uL]	7.1 ± 1.9	7.9 ± 1.1
Red blood cells [Million/uL]	4.8 ± 0.3	5.2 ± 0.7
Hemoglobin [g/dL]	14.8 ± 1.2	12.9 ± 0.3
Hematocrit [%]	43.7 ± 3.2	38.7 ± 2.0
Platelet count [Thous/uL]	247.8 ± 77.8	302.6 ± 21.4
Glucose [mmol/L]	6.2 ± 1.2	5.2 ± 0.2
Blood urea nitrogen [mmol/L]	6.5 ± 2.0	7.2 ± 0.3
Creatinine [µmol/L]	74.8 ± 13.9	73.3 ± 7.2
Sodium [mEq/L]	142.4 ± 1.9	139 ± 0.6
Potassium [mEq/L]	4.3 ± 0.3	4.1 ± 0.3
Chloride [mEq/L]	102.9 ± 2.0	100.7 ± 1.1
Total bilirubin [µmol/L]	12.3 ± 8.5	9.3 ± 1.2
T-cholesterol [mmol/L]	4.8 ± 1.0	3.9 ± 0.3
High-density lipoprotein cholesterol [mmol/L]	1.3 ± 0.3	2.0 ± 0.2
Low-density lipoprotein cholesterol [mmol/L]	2.9 ± 0.9	1.8 ± 0.08
Triglyceride [mmol/L]	1.2 ± 0.4	1.7 ± 0.1
C-reactive protein [mg/L]	2.2 ± 1.6	1.0 ± 0.2
Thyroid-stimulating hormone [µIU/mL]	1.6 ± 1.3	0.4 ± 0.1
Alanine transaminase [U/L]	14.9 ± 4.5	20 ± 4.1
Gamma-Glutamyl Transpeptidase [IU/L]	25.9 ± 12.8	23.3 ± 6.2
Erythrocyte sedimentation rate	14.1 ± 1.4	7.0 ± 0.8
**Urine**		
Urine color	yellow/dark amber (24/3)	yellow
Clarity	clear/cloudy (22/5)	clear
Acidity	normal/acidic(17/10)	normal
Specific gravity	1.020 ± 0.02	1.020 ± 0.02
Glucose	negative	negative
Ketones	negative	negative
Nitrates	negative	negative
Bilirubin	negative	negative
Urobilirubin	negative	negative
Blood	≤3 red blood cells	≤3 red blood cells
Red blood cells	≤2 RBCs/hpf	≤2 RBCs/hpf
White blood cells	≤2–5 WBCs/hpf	≤2–5 WBCs/hpf
Protein	≤150 mg/dL	≤150 mg/dL
Squamous epithelial cells	negative	negative
Casts	negative	negative
Crystals	negative	negative
Bacteria	none/present (22/5)	none
Yeast	none	none
